# The A736V TMPRSS6 Polymorphism Influences Hepatic Iron Overload in Nonalcoholic Fatty Liver Disease

**DOI:** 10.1371/journal.pone.0048804

**Published:** 2012-11-05

**Authors:** Luca Valenti, Raffaela Rametta, Paola Dongiovanni, Benedetta M. Motta, Elena Canavesi, Serena Pelusi, Edoardo A. Pulixi, Anna L. Fracanzani, Silvia Fargion

**Affiliations:** Department of Pathophysiology and Transplantation, Section Internal Medicine, Università degli Studi, Fondazione Ca' Granda IRCCS Ospedale Maggiore Policlinico, Milano, Italy; University of Verona, Ospedale Civile Maggiore, Italy

## Abstract

**Background & Aims:**

Hepatic iron accumulation due to altered trafficking is frequent in patients with nonalcoholic fatty liver disease (NAFLD), and is associated with more severe liver damage and hepatocellular carcinoma. The p.Ala736Val TMPRSS6 variant influences iron metabolism regulating the transcription of the hepatic hormone hepcidin, but its role in the pathogenesis of iron overload disorders is controversial. Aim of this study was to evaluate the whether the TMPRSS6 p.Ala736Val variant influences hepatic iron accumulation in a well-characterized series of Italian patients with histological NAFLD.

**Methods:**

216 patients with histological NAFLD. TMPRSS6 and HFE variants were assessed by allele specific PCR, liver histology by the NAFLD activity score and Perls' staining for iron.

**Results:**

Homozygosity for the p.736Val allele previously linked to higher hepcidin did not influence transferrin saturation (TS), but was associated with lower hepatic iron stores (p = 0.01), and ferritin levels (median 223 IQR 102–449 vs. 308 IQR 141–618 ng/ml; p = 0.01). Homozygosity for TMPRSS6 p.736Val was nearly associated with lower ballooning (p = 0.05), reflecting hepatocellular damage related to oxidative stress. The influence of TMPRSS6 on hepatic iron accumulation was more marked in patients negative for HFE genotypes predisposing to iron overload (p.Cys282Tyr + and p.His63Asp +/+; p = 0.01), and the p.736Val variant was negatively associated with hepatic iron accumulation independently of age, gender, HFE genotype, and beta-thalassemia trait (OR 0.59, 0.39–0.88).

**Conclusions:**

The p.Ala736Val TMPRSS6 variant influences secondary hepatic iron accumulation in patients with NAFLD.

## Introduction

Liver fat deposition related to systemic insulin resistance and the metabolic syndrome defines nonalcoholic fatty liver disease (NAFLD) [1], [2]. In susceptible individuals NAFLD is associated with oxidative hepatocellular damage, inflammation, and activation of fibrogenesis, i.e. non-alcoholic steatohepatitis (NASH) [3], [4], with potential progression towards cirrhosis and hepatocellular carcinoma [5], [6]. Due to the epidemic of obesity and the metabolic syndrome, NAFLD is now the most frequent liver disease and the leading cause of altered liver enzymes in Western countries [7], [8], and it is projected to become the leading cause of end-stage liver disease and hepatocellular carcinoma.

Hyperferritinemia and mild iron overload are strongly associated with NAFLD [9], [10], [11], [12], [13], [14], [15], being detected in about 30% of unselected patients [11], [16], [17], [18], [19], [20]. In patients with steatosis, body iron stores have been linked to a heightened risk of metabolic complications [21], [22], [23], faster progression of cardiovascular damage [24], [25], [26], [27], more severe liver inflammation and fibrosis [9], [10], [11], [15], [28], [29], [30], and hepatocellular carcinoma [31]. In addition, iron depletion reduced insulin resistance and indices of liver necrosis in controlled studies [15], [17]. Differently from genetic iron overload disorders [32], NAFLD and the metabolic syndrome are characterized by preserved upregulation of the iron hormone hepcidin [15], [33], which inhibits iron absorption and recycling by binding and inactivating the cellular iron exporter Ferroportin-1 [34]. Therefore, the pathogenesis of iron accumulation in NAFLD has been related to altered cellular iron export associated with steatosis, hepatic inflammation, and IR [15], [35].

Genetic factors influencing the regulation of the iron hormone hepcidin, such as mutations in the *HFE* gene of hereditary hemochromatosis, contribute to the susceptibility to iron accumulation in NAFLD [11], [15], [18]. We have also shown that *beta-globin* mutations represent the best predictor of parenchymal iron overload in Italian patients with NAFLD, and are associated with a two-fold higher risk of severe fibrosis [12], linking iron overload to progressive liver disease.

The *Trans-membrane protease serine 6* (*TMPRSS6*) gene encodes for enzymes matriptase-2, which cleaves the membrane-bound hemojuvelin, a bone morphogenetic protein (BMP) coreceptor required for hepcidin expression in the liver, thereby decreasing hepcidin transcription [36]. Rare loss-of-function mutations in *TMPRSS6* cause iron-refractory iron deficiency anemia (IRIDA) by upregulating hepcidin due to the inability to cleave hemojuvelin [37], whereas common *TMPRSS6* polymorphisms are a risk factor for iron-deficiency anemia. The strongest association was observed for *TMPRSS6* rs855791 C>T polymorphism in exon 17, a nonsynonymous substitution encoding for the p.Ala736Val variant that reduces the ability of the enzyme to cleave hemojuvelin and therefore to inhibit hepcidin transcription [38], thereby influencing transferrin saturation (TS), and erythropoiesis [37], [39], [40], [41], [42]. We recently reported that the p.Ala736Val variant influences the penetrance and expression of hereditary hemochromatosis [43]. However, the relationship between *TMPRSS6* genotype and the expression of hemochromatosis is still controversial, and no data are available on the effect on iron overload disorders not related to hepcidin dysregulation.

Aim of this study was to evaluate the whether the TMPRSS6 p.Ala736Val variant influences hepatic iron accumulation in a well-characterized series of Italian patients with histological NAFLD.

## Materials and Methods

### Patients

We considered 216 out of 274 Italian patients (79%) with biopsy proven NAFLD reported in a previous paper [12], without phenotypically expressed hereditary hemochromatosis, whose DNA samples and complete clinical data were available, and who gave written informed consent. The clinical features of the patients included are not significantly different from those of subjects not included in this study (not shown). Other causes of liver disease were excluded, including increased alcohol intake (>30/20 g/day for M/F), as confirmed by at least one family member and carboxydesialylated transferrin determination (<1.5%), the p.Cys282Tyr +/+ *HFE* genotype typical of hereditary hemochromatosis, hereditary iron overload due to Ferroportin-1 mutations, alpha1-antitrypsin deficiency (PiZ/Z, PiZ/S), chronic viral and autoimmune hepatitis, Wilson disease, and celiac disease. BMI and metabolic parameters, including glucose and lipid levels, ferritin, and liver enzymes (AST, ALT, GGT), and evaluation of acquired causes of iron overload, were determined in all patients. Serum hepcidin levels, available in a subset of patients (n = 55), were determined as previously described [44] for the evaluation of the relationship between iron and vascular damage in the same cohort [24], [27]. Hyperferritinemia has been defined for values of serum ferritin greater than 240 ng/ml in women and 320 ng/ml in men (upper reference value for the normal population in our laboratory) [12].

We also considered 271 healthy blood donors with normal iron parameters (serum ferritin and transferrin saturation (TS) at first blood donation), liver enzymes (AST, ALT, GGT), and metabolic parameters (glucose, triglycerides) as controls, all of Italian ancestry (Milan area). The study was approved by the institutional Review Board of the Fondazione Ca' Granda IRCCS, and it has been conducted according to the principles expressed in the Declaration of Helsinki. All evaluated subjects signed an informed consent. Demographic and clinical features of the subjects included are shown in table 1.

**Table 1:** Demographic, clinical, and genetic features of 216 Italian patients with NAFLD with available re-evaluation of histological siderosis and of 271 healthy controls with normal iron parameters, liver function tests, and metabolic parameters.

	NAFLD patients	Healthy controls
Number	216	271
Sex F	49 (18)	56 (21)
Age years	49±12*	45±12
BMI Kg/m^2^	27±4*	26±3
Hb g/dl	14±2	14±2
Total cholesterol mg/dl	206±40	193±33
HDL cholesterol mg/dl	47±14*	57±13
Triglycerides mg/dl	151±89*	88±46
Glucose mg/dl	99±24*	67±33
HOMA-IR	4.1±3.2*	1.2±0.2
ALT UI/ml	54±40*	23±9
GGT UI/ml	72±79*	21±13
TS%	34±12	33±9
Ferritin ng/ml	286 {138–585}	37 {22–68}
Fibrosis stage F 2–4	38 (18)	-
*HFE* genotype		
p.Cys282Tyr/His63Asp	10 (5)	2 (1)
p.Cys282Tyr +/wt	15 (7)	7 (2)
p.His63Asp/His63Asp	9 (4)	9 (3)
p.His63Asp/wt	54 (25)	76 (28)
wt/wt	128 (59)	178 (66)
Beta-thalassemia trait	22 (10)*	0∧
*TMPRSS6* p.Ala736Val		
Ala/Ala	82 (38)	88 (32)
Ala/Val	101 (47)	137 (51)
Val/Val	33 (15)	46 (17)

(): % values; BMI: body mass index; Hb: hemoglobin concentration; TS%: transferrin saturation %; wt: wild-type variant;

* p<0.05 vs. healthy controls;

∧ beta-thalassemia trait was a *criterium* for exclusion from blood donation.

### Histological assessment

Tissue sections were stained with hematoxylin and eosin, impregnated with silver for reticulin framework, and stained with trichrome for collagen. One expert pathologist unaware of clinical and genetic data reviewed all biopsies for fibrosis stage and the presence and pattern of liver siderosis. The presence of NASH was assessed according to Kleiner et al. [45]. The minimum biopsy size was 1.7 cm and the number of portal areas 10. Histological re-evaluation of liver siderosis was performed by one expert pathologist according to Deugnier [46].

When detected, hepatic iron accumulation was defined as hepatocellular or non-parenchymal according to the prevalent distribution pattern of siderosis [12].

### Genetic analysis

DNA was extracted from peripheral blood by the phenol-chloroform method. Success rate in extracting DNA was 100% for each study group. *HFE* genotype (p.Cys282Tyr and p.His63Asp variants), and the *TMPRSS6* rs855791 C>T polymorphism, (p.Ala736Val variant) were determined by sequence allele specific PCR, as previously described [12], [43]. Random samples were confirmed by direct sequencing. Quality controls were performed to verify the reproducibility of the results. Valid genotypic data were obtained for 100% of subjects analyzed.

### Statistical analysis

Values are expressed as means±SD or median {interquartile range} according to distribution. Mean values were compared by Anova or Wilcoxon (as required), frequencies by Chi-square test and Cochrane-Armitage test for trend, when appropriate. The association of the *TMPRSS6* p.Ala736Val variant with iron overload and the severity of liver disease was evaluated by logistic regression analysis, considering as independent variables demographic and anthropometric features and genetic variables significant at univariate analysis. P values were considered significant when ≤0.05 (two-tailed). The sample size had >90% power (p<0.05) of detecting 2-fold variation in risk of hepatic iron accumulation. Analyses were carried out with JMP 6.0 statistical analysis software (SAS Institute Inc, Cary, NC).

## Results

### Clinical and genetic features of patients and controls

As expected, patients with NAFLD had higher BMI, insulin resistance, dyslipidemia, ferritin levels, and liver enzymes compared to healthy controls (table 1). There was a nonsignificant trend for an increased prevalence of the p.Cys282Tyr *HFE* mutations in patients, whereas the prevalence of the beta-thalassemia trait was significantly higher in patients than in controls, but it represented and exclusion *criterium* for the control group. The frequency distribution of the p.Ala736Val *TMPRSS6* variant was in Hardy-Weinberg equilibrium and not significantly different from that of healthy controls with normal iron parameters (table 1; p = ns), suggesting that, as expected, *TMPRSS6* genotype is not a risk factor for NAFLD.

### Effect of TMPRSS6 p.Ala736Val in patients with NAFLD

Clinical features of NAFLD patients subdivided according to the p.Ala736Val *TMPRSS6* status are shown in table 2. Homozygosity for the p.736Val allele (henceforth 736V/V), previously linked to higher hepcidin release, did not influence Hb levels. However, in line with data observed in the general population, 736V/V patients without the beta-thalassemia trait had significantly lower MCV values (table 2). The 736V/V status was associated with lower ferritin levels (median 223 IQR 102–449 vs. 308 IQR 141–618 ng/ml; p = 0.01), but not with lower TS.

**Table 2:** Clinical features of 216 Italian patients with NAFLD subdivided according to the p.Ala736Val *TMPRSS6* status.

*TMPRSS6* p.Ala736Val	Ala/Ala	Ala/Val	Val/Val	p value
Number	82	101	33	
Sex F	12 (15)	18 (18)	8 (24)	0.48
Age years	49±11	48±12	48±11	0.93
BMI Kg/m^2^	26.9±2.7	26.9±3.9	26.0±2.8	0.35
Hb g/dl°	14.7±1.2	14.7±1.3	14.6±1.5	0.81
MCV°	90±4	90±6	88±5*	0.10
TS %	34±16	33±12	35±14	0.62
Ferritin ng/ml	290 {141–590}	328 {135–628}	223 {102–449}*	0.08
Total cholesterol mg/dl	200±39	202±40	199±44	0.92
HDL cholesterol mg/dl	46±13	47±15	43±12	0.40
Triglycerides mg/dl	145±82	156±90	140±77	0.55
Glucose mg/dl	97±22	97±29	96±17	0.94
HOMA-IR	4.0±3.2	4.3±3.6	4.0±3.2	0.88
ALT UI/ml	49±37	54±44	58±35	0.42
GGT UI/ml	72±87	79±84	76±87	0.86
Hepatocellular iron positive	26 (32)	22 (22)	6 (18)	0.08
Nonparenchymal iron positive	31 (38)	30 (30)	7 (10)	0.06
Positive iron stores	39 (48)	33 (33)	9 (27)	0.01
Steatosis >33%	20 (24)	16 (16)	7 (21)	0.46
Necroinflammation score >1	4 (5)	2 (2)	1 (3)	0.80
Ballooning score >1	9 (11)	8 (8)	0	0.05
Fibrosis stage<1	17 (21)	16 (16)	5 (15)	0.38

(): % values; BMI: body mass index; Hb: hemoglobin concentration; MCV mean corpuscular volume;

° evaluated in cases negative for the beta-thalassemia trait (72 Ala/Ala, 91 Ala/Val, 31 Val/Val);

* p<0.05 for Val/Val vs. Ala/Ala and Ala/Val (recessive model).

Supporting a negative association with iron accumulation, 736V/V was also associated with a lower prevalence of detectable hepatic iron stores (p = 0.01).

**Figure 1:**
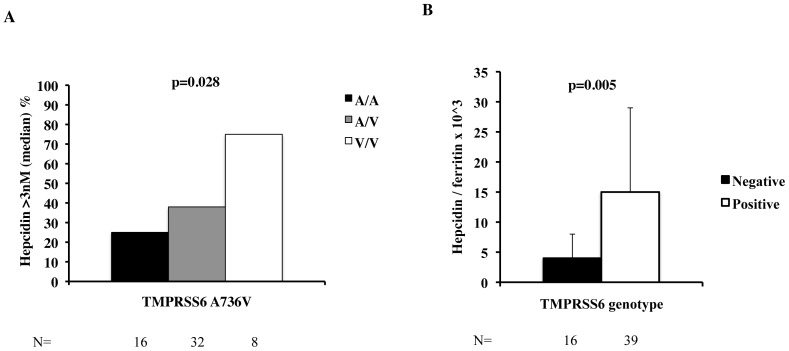
Effect of *TMPRSS6* p.A736V genotype on hepcidin levels (hepcidin <3 nM, median value, panel A) and the hepcidin/ferritin ratio (panel B) in 55 patients with NAFLD (25.5% of the whole series).

In the 55 patients (16 Ala/Ala, 31 Ala/Val, 8 Val/Val) with available hepcidin-25 measurement at the time of liver biopsy, the p.Ala736Val variant was associated with an increasing prevalence of high hepcidin (>3 nM, median value) with increasing number of 736 V alleles, despite lower iron stores (p = 0.028 for trend; figure 1A). Furthermore, H/F was higher in the 736Val positive than in negative patients (p = 0.005; figure 1B). However, this analysis is limited by lack of available data in the majority of patients considered.

The 736V/V was associated with decreased prevalence of severe hepatocellular ballooning (p = 0.05), reflecting hepatocellular damage related to oxidative stress, despite similar steatosis, necroinflammation, and fibrosis stage (table 2).

### Interaction between TMPRSS6 and HFE genotypes and effect of the beta thalassemia trait on iron metabolism in NAFLD

*HFE* genotypes at risk for iron overload (p.Cys282Tyr positive and His63Asp/His63Asp, n = 34) were associated with higher TS (42±12 vs. 32±12; p = 0.0004) and hepatic iron deposition (23/34, 68% vs. 58/182, 32%; p = 0.0002), and with higher serum ferritin (458 {240–622} vs. 237 {134–574} ng/ml; p = 0.08).

**Figure 2:**
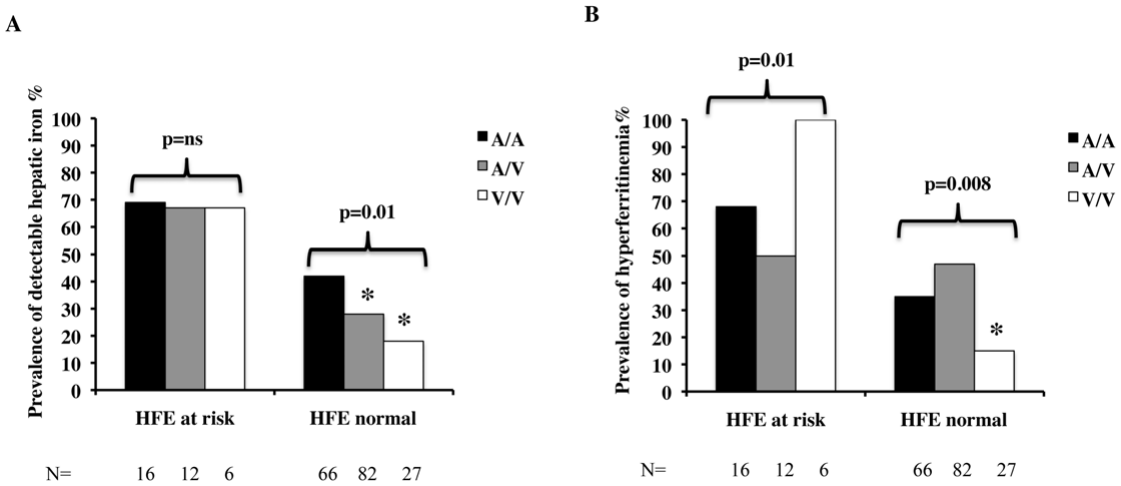
Combined effect of the *HFE* and *TMPRSS6* genotypes on iron status in 216 Italian patients with NAFLD. A) Prevalence of detectable hepatic iron deposition B) Prevalence of increased ferritin levels (>320/240 ng/ml in M/F). p values are for trend across *TMPRSS6* genotypes in patients carrying *HFE* genotypes at risk or not. * p<0.05 vs. *HFE* genotypes at risk.

*TMPRSS6* p.Ala736Val status influenced hepatic iron deposition in patients negative, but not in those positive for *HFE* genotypes at risk for iron overload, whereas *HFE* genotypes at risk influenced hepatic iron accumulation in patients positive for the p.736Val TMPRSS6 variant (figure 2A). In contrast, p.Ala736Val status influenced ferritin levels in both patients with and without *HFE* genotypes at risk, although in opposite direction (figure 2B). In patients without *HFE* genotypes at risk, concordantly with decreased iron stores, the prevalence of hyperferritinemia was lower in 736V/V subjects, whereas in the presence of *HFE* genotypes at risk 736V/V was associated with increased ferritin. TS was not influenced by *TMPRSS6* genotype neither in patients positive of negative for *HFE* genotypes at risk (not shown).

Besides with red cell parameters (MCV 67±8 vs. 90±5 fl, p<0.0001; Hb 12.5±1.6 vs. 14.7±1.3 g/dl, p<0.0001), the beta-thalassemia trait, detected in 22 patients (10%), was associated with higher ferritin (531, 132–858 vs. 252, 131–568, p = 0.01), TS (38±10 vs. 33±12%, p = 0.03), lower H/F (2, 1–12 vs. 7, 2–18×10^3^, p = 0.019), and hepatic iron accumulation (15/23, 65% vs. 66/193, 34%, p = 0.005).

### Determinants of hepatic iron accumulation

**Figure 3:**
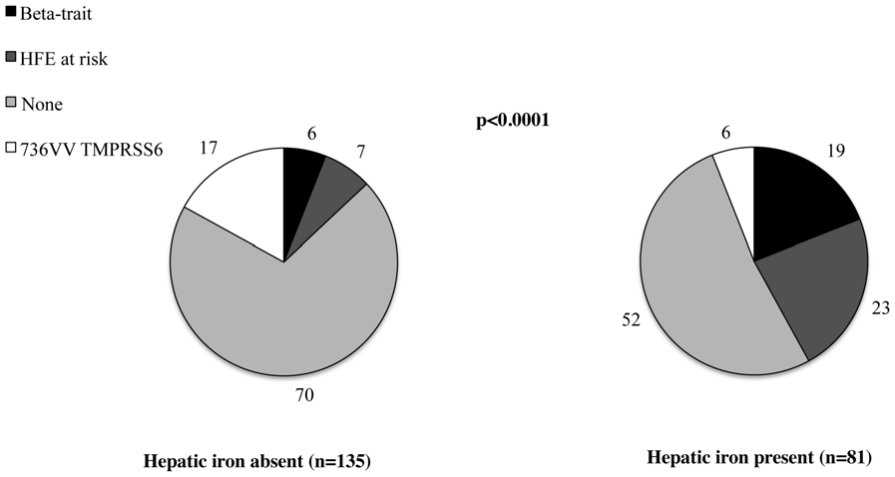
Frequency distribution of genetic factors influencing iron metabolism in patients with and without hepatic iron accumulation. Patients positive for the beta-thalassemia trait were classified as “Beta-trait”, next if negative for “Beta-trait” and positive for *HFE* genotypes at risk as “HFE at risk”, and among the remaining patients those homozygous for the 736V/V TMPSS6 as “736VV”, and the rest as “none”.

The frequency distribution of genetic risk factors in patients with and without hepatic iron accumulation is presented in figure 3. The prevalence of beta-thalassemia trait and of *HFE* genotypes at risk was higher, whereas that of 736V/V *TMPRSS6* was lower in patients with hepatic iron (p<0.0001).

The clinical and genetic parameters associated with hepatic iron accumulation at univariate analysis are presented in table 3. The independent determinants of hepatic iron accumulation are presented in table 4. Iron parameters were excluded from the analysis in order to test the independent effect of genetic factors after correction for demographic and anthropometric features. The *TMPRSS6* p.736Val allele was negatively associated with hepatic iron accumulation independently of age, gender, the presence of HFE genotypes at risk, and the beta-thalassemia trait (OR 0.59, 95% confidence interval 0.39–0.88, per each p.736Val allele).

**Table 3:** Relationship between clinical and genetic variables considered in the study with hepatic iron accumulation in 216 patients with NAFLD (univariate analysis).

	Iron present(n = 81)	Iron absent(n = 135)	p value
Age	50.2±11	47.4±11	0.040
Male gender	72 (89)	106 (78)	0.046
BMI Kg/m^2^	26.2±2.6	27.2±3.6	0.018
Total cholesterol mg/dl	195±37	204±41	0.11
HDL cholesterol mg/dl	45±13	46±14	0.56
Triglycerides mg/dl	120 {92–165}	135 {92–185}	0.18
HOMA-IR	3.2 {2.1–4.3}	3.2 {2.3–5.2}	0.18
ALT IU/ml	48±45	56±36	0.18
GGT IU/ml			
CRP mg/dl	0.1 {0–0.39}	0.1 {0–0.45}	0.83
TS%	36±11	32±12	0.016
Ferritin ng/ml	536 {273–724}	184 {116–384}	<0.0001
*HFE* genotypes at risk	23 (28)	11 (8)	0.0002
Beta-thalassemia trait	15 (19)	8 (6)	0.006
*TMPRSS6* p.Ala736Val genotype			0.018
Ala/Ala	39 (48)	43 (32)	
Ala/Val	33 (41)	68 (50)	
Val/Val	9 (11)	24 (18)	

ALT: alanine aminotransferases; BMI: body mass index; CRP: C reactive protein; TS%: transferrin saturation %; *HFE* genotypes at risk: p.Cys282Tyr positive and p.His63Asp/His63Asp.

**Table 4:** Independent determinants of hepatic iron accumulation in 216 patients with NAFLD at multivariate logistic regression analysis.

	OR	95% c.i.	p value
Age	1.02	1.01–1.04	0.005
Male gender	3.50	1.46–9.36	0.004
BMI Kg/m^2^	0.94	0.88–1.00	0.044
*HFE* genotypes at risk	4.87	2.18–11.40	<0.0001
Beta-thalassemia trait	2.95	1.14–8.07	0.025
*TMPRSS6* p.736Val allele	0.59	0.39–0.88	0.0099

95% c.i.: 95% confidence interval; *HFE* genotypes at risk: p.Cys282Tyr positive and p.His63Asp/His63Asp.

In this model, the effect of the p.Ala736Val *TMPRSS6* variant was adjusted for age, gender, BMI and the other genetic factors.

## Discussion

In this paper, we evaluated whether the *TMPRSS6* p.Ala736Val variant influencing hepcidin transcription and iron status in the general population [38], [39], [40], [41], [47], [48], affects hepatic iron overload in patients with NAFLD. The study was prompted by evidence indicating that iron overload unrelated to hepcidin dysregulation is frequently observed in patients with NAFLD [15], is favored by genetic factors [11], [12], and is associated with the severity of hepatic and vascular disease [11], [14], [29], [49]. *TMPRSS6* genotype has been shown to influence iron metabolism in animal models of hereditary hemochromatosis and beta-thalassemia [50], [51], but the effect on iron overload in patients with hemochromatosis is still controversial [43], and no data were available for the effect of *TMPRSS6* genotype in models of iron overload where hepcidin upregulation is preserved 15,33.

In line with the experimental hypothesis, the results of the present study suggest that the p.Ala736Val variant influences the susceptibility to iron accumulation in NAFLD, even if, as expected, is not a risk factor for NAFLD. Indeed, the p.736Val “high hepcidin” allele was associated with lower ferritin levels, and most importantly with the absence of hepatic iron accumulation, i.e. with direct evidence of iron overload. However, differently from what was observed in the general population, *TMPRSS6* genotype did not influence TS, which on the other hand was modulated by *HFE* mutations and the beta-thalassemia trait. Notably, the negative association with the presence of hepatic iron accumulation was independent of demographic features, such as age and gender, and of other genetic factors (*HFE* mutations and the beta thalassemia trait). Although hepcidin was measured by mass spectrometry in a minority of subjects (only about one quarter), the available data suggest that the mechanism linking the p.736Val allele to protection from iron overload is related to increased serum hepcidin levels (despite lower iron stores), which is consistent with data indicating that this genetic variant positively influences hepcidin transcription [38]. It can be hypothesized that the effect of the p.736 V variant on hepcidin levels is magnified in NAFLD patients by the induction of hepcidin transcription due to overweight, liver steatosis, and subclinical inflammation [15]. However, given the aforementioned limitations, these data should be interpreted with caution. Therefore, the main results of the present manuscript is that *TMPRSS6* p.Ala736Val variant influences iron accumulation even in secondary iron overload disorders which are not caused by a deficit in the upregulation of hepcidin in response to iron, such as hereditary hemochromatosis. The *TMPRSS6* p.Ala736Val variant may thus represent a modifier of iron overload in other conditions, besides in NAFLD, characterized by altered iron trafficking such as chronic inflammatory diseases, the anemia of chronic diseases, and possibly some neurologic diseases characterized by altered iron compartmentalization [32].

Interestingly, the effect of *TMPRSS6* on the presence of detectable hepatic iron was more marked in subjects negative for *HFE* genotypes predisposing to iron overload, suggesting that *HFE* mutations may have a dominant effect at least in the NAFLD setting, but the study was not powered enough to test the interaction between these genetic factors.

An interesting collateral finding was that, as expected based on the previous results, 736V/V homozygosity was associated with a lower prevalence of hyperferritinemia in patients without *HFE* at risk, but was paradoxically associated with higher ferritin levels without altering hepatic iron stores in patients with *HFE* mutations increasing iron absorption. These data suggest that the single evaluation of traditional serum iron parameters may not be sufficient to study the interaction between *HFE* and *TMPRSS6* in the regulation of iron metabolism. Indeed, *HFE* and *TMPRSS6* genotypes influenced different iron parameters (TS and ferritin respectively), and the phenotypes were modified when mutations occurred together, suggesting that *HFE* and *TMPRSS6* variants alter iron trafficking by partially different mechanisms. It could be speculated that the *TMPRSS6* p.736Val variant promotes a shift in iron balance towards macrophages and ferritin secretion by facilitating hepcidin release and iron fluxes towards macrophages. This would lead to an overestimation of iron stores, in particular in subjects with *HFE* mutations predisposing to increased iron absorption by mechanisms partially independent of hepcidin [41]. This hypothesis should be taken into account when evaluating the effect of *TMPRSS6* on iron overload disorders, leaving as the most reliable markers iron removed to depletion and the direct assessment of hepatic iron.

Notwithstanding, in the overall series of patients the negative association (with borderline significance) between the p.736Val variant and the presence of detectable hepatic iron was paralleled by decreased levels of hepatocellular ballooning, a typical manifestation of oxidative stress in NAFLD [52], which is possibly related to iron status. However, these associations, that need to be confirmed in larger studies including patients from different geographic areas, did not translate into increased severity of liver fibrosis, possibly due to the limited number of subjects with advanced liver disease included in this study.

### Conclusions

In conclusion, the p.Ala736Val *TMPRSS6* variant influences hepatic iron accumulation in patients with NAFLD, likely by influencing hepcidin levels, indicating that *TMPRSS6* genotype affects iron accumulation related to altered iron trafficking.
